# A Comparison of the Contractile Properties of Myometrium from Singleton and Twin Pregnancies

**DOI:** 10.1371/journal.pone.0063800

**Published:** 2013-05-06

**Authors:** Peter Turton, Sarah Arrowsmith, Jonathan Prescott, Celia Ballard, Leanne Bricker, James Neilson, Susan Wray

**Affiliations:** 1 Department of Cellular and Molecular Physiology, Institute of Translational Medicine, University of Liverpool, Liverpool, United Kingdom; 2 Liverpool Women's NHS Foundation Trust, Liverpool, United Kingdom; 3 Department of Women's and Children's Health, Institute of Translational Medicine, University of Liverpool, United Kingdom; University of Nevada School of Medicine, United States of America

## Abstract

**Objective:**

Over half of twin pregnancies in US and UK deliver prematurely but the reasons for this are unclear. The contractility of myometrium from twin pregnancies has not been directly investigated. The objective of this research was to determine if there are differences in the contractile activity and response to oxytocin, between myometrium from singleton and twin pregnancies, across a range of gestational ages. Furthermore, we wished to determine if contractile activity correlates with increasing level of stretch, using neonatal birth weights as a marker of uterine stretch.

**Methods:**

This was an *in vitro*, laboratory based study of myometrial contractility in women pregnant with one or two babies, using biopsies obtained from non-labouring women undergoing Caesarean section. Spontaneous, oxytocin-stimulated and depolarization induced contractile activity was compared.

**Results:**

Direct measurements of myometrial contractility under controlled conditions show that the frequency of contractions and responses to oxytocin are significantly increased in twins compared to singletons. The duration of contraction however was significantly reduced. We find that contractile activity correlates with increasing levels of stretch, using neonatal birth weights as a surrogate for uterine stretch, with response to oxytocin being significantly positively correlated with birth weight.

**Conclusions:**

We have found significant differences in contractile properties between myometrium from singleton and twin pregnancies and that increasing uterine stretch can alter the contractile properties of myometrium. We discuss the implication of these findings to preterm delivery and future studies.

## Introduction

Twin pregnancies are known to occur spontaneously in one in every eighty pregnancies. Amongst the many problems facing multiple pregnancy, preterm labour and delivery remains the commonest, with over half delivering before 37 weeks [1], largely due to spontaneous preterm labours [2]. Premature activation of contractility underlies spontaneous preterm delivery [1]. Despite this and the very high rate of preterm labour, there is limited research into how contractile activity in twin pregnancy differs from singletons [2]. Home monitoring studies suggest that twin pregnancies [3], [4], and triplets [4] have a higher frequency of contractions, after 35 weeks. The increasing frequency has been shown to correlate with increasing cervical changes prior to labour [5]. How well the increased frequency of contractions reported in twin pregnancies correlates or predicts preterm labour is however unclear: Some studies show that an elevated rate of contractions in twin pregnancies does increase the risk of preterm labour [5], and uterine monitoring studies show that twin pregnancies that go on to deliver preterm, have a contraction frequency that is significantly greater than twins that deliver at term [6]. Other studies however concluded that the increased frequency was not predictive of preterm labour [4], [7].

It is unclear as to whether increased stretching of myometrium is a responsible mechanism for preterm labour in twin pregnancies. Using ultrasound evaluation, the myometrial thickness of the lower uterine segment is thinner in preterm twin pregnancies than term twin or singleton pregnancies, suggesting that greater distension leads to poor adaptation of the lower segment [8]. Stretch increases the expression of oxytocin receptors, cytokines and gap junctions, the effects of these changes on contractility are yet to be determined, and only one study has compared their expression levels in twins and singletons, and found little difference [9]. No direct comparison of myometrial responsiveness to oxytocin, testing the hypothesis that twins will have a greater contractile response, has been made.

Previous work from our group has shown that *in vitro* myometrial contractility can reflect *in vivo* clinical activity and labour outcome [10], [11]. The question therefore arises whether *in vitro* activity of myometrium from twins is different to that of singletons, making it a useful model for mechanistic studies focussed on risk of preterm labour.

There are no studies directly investigating contractility of myometrium from twin pregnancies and comparing it with that from singletons. The aims of our study are therefore in singleton and twin pregnancies to: (1) Directly compare under standardised conditions their inherent myometrial contractile parameters, (2) Determine the effects of gestation on these contractile parameters, (3) Investigate the response of the myometrium to oxytocin stimulation and (4) Investigate the relationship between *in vivo* myometrial stretch and contractile parameters.

## Materials and Methods

### Ethics statement

This study was given favourable ethical opinion and was approved by the North West (Liverpool East) Research Ethics Committee (REC Ref; 09/H1002/64) and by the Research and Development Director of Liverpool Women's NHS Foundation Trust, Liverpool, UK. All women provided written informed consent for the collect of samples and subsequent analysis.

### Tissue collection and preparation

We recruited 83 women undergoing elective lower segment Caesarean section at Liverpool Women's Hospital, Liverpool, UK. At operation, a full thickness biopsy (around 2 cm long, 1 cm wide and 1 cm thick) was obtained from the upper lip of the lower uterine segment incision [12] after the safe delivery of the fetus and placenta. Care was taken to avoid any scar tissue from women having repeat Caesarean section. All biopsies were immediately placed into Hanks Balanced Salt Solution, stored at 4°C and used within 12 hours of collection. The women were not in labour and had either a singleton or twin pregnancy. Exclusions factors were women with serious medical illness, taking anti-hypertensive medication or pregnant with more than two babies. To limit the effects of the differences in gestational age between term singleton and twin pregnancies, any biopsies obtained from singleton pregnancies >269 days gestation (38 weeks and 3 days) were excluded. This means that all term samples from both groups came from a narrow 10 day window of 259 to 269 days gestation.

Clinical data relating to each pregnancy was obtained retrospectively from the hospital database (Meditech), looking for gestational age, maternal age, parity, reason for CS, neonatal birth weight(s) at delivery and maternal BMI, recorded at the time of pregnancy booking (10–12 weeks). In the case of the twin pregnancies, the birth weight of each neonate was combined.

Reasons for elective CS in singletons were; previous CS (n = 18), breech (n = 6) placenta previa (n = 1), maternal request (n = 1), fetal abnormality (n = 7; Intra Uterine Growth Restriction (IUGR) (n = 1), cardiotocography (CTG) outside normal limits (n = 6)) and previous difficult vaginal delivery (n = 2). In twins, reasons for CS were; previous CS (n = 4), breech (n = 10), placenta previa (n = 1), multiple pregnancy/maternal request (n = 24), fetal abnormality (n = 5; IUGR (n = 3), CTG outside normal limits (n = 2)) and previous difficult vaginal delivery (n = 4). None of the samples were from women having preterm rupture of membranes (PROM). Biopsies were cleared of endometrium, excess blood and any fetal membranes present. Longitudinal strips of myometrium (5 mm long, 2 mm wide and 1 mm thick) were dissected from the biopsies. Aluminium clips were attached to each end [13] and the muscle strips were mounted to a tension transducer in a 1 ml chamber bath. The strips were superfused with physiological salt solution (in mM: 154 NaCl, 5.6 KCl, 1.2 MgSO_4_, 7.8 glucose, 10.9 HEPES and 2.0 CaCl_2_,) at a rate of 1.5 ml/min at pH 7.4 and maintained at a temperature of 37°C, until spontaneous contractile activity commenced [14]. All strips were placed under a resting tension of 2 mN to ensure the amount of stretch applied was standardised across experiments.

### Contractile measurements

After strips began to contract, a control period was established of between 4–6 contractions, each of a similar amplitude and frequency. In this control period, measurements of mean amplitude, duration and frequency of contractions, as well as total integral of force (measured as area under the curve, AUC) were taken [15], [16].

Following this period of control activity, one of two experiments was performed. In the first (n = 49: n = 32 singletons, n = 17 twins), a depolarizing stimulus, using high potassium salt solution (elevated to 40 mM by isosmotic substitution of NaCl for KCl) superfused through the chamber for 2 minutes, was given to obtain a measure of maximum force [17]. In the second experiment (n = 39: n = 17 singletons, n = 22 twins), a 10 nM solution of oxytocin was superfused through the chamber for 20 minutes.

### Analysis

Unless stated otherwise, data refer to spontaneous contractions. For analysis of the differences in contractility patterns between the pregnancy groups, we compared peak amplitude above baseline (expressed as force in mN), frequency of contractions (determined over at least 4 contractions and normalised as rate per 10 minutes), contraction duration (determined at 50% of peak amplitude and averaged over 4 contractions) and AUC in arbitrary units (normalised to unit time).

For comparison of high K-stimulated contraction (first experiment), the maximum amplitude in high K was assigned as 100%. The mean amplitude of the control period (average of at least 4 contractions) was normalised to the high K contraction (expressed as percentage of amplitude in high K). For completeness, we also measured AUC for 2 minutes under high K. In the second experiment, the amplitude of the tonic contraction with oxytocin was divided by the mean amplitude of contraction during the control period (100%) to give the percentage increase in amplitude under oxytocin stimulation. The percentage increase in AUC under oxytocin compared to control activity was also calculated.

For each of the contractile measurements, comparisons were made between groups in three ways; in the first, an overall comparison between singleton and twin pregnancy myometrium was performed. In the second, the singleton and twin pregnancy myometrium groups were subdivided into preterm (before 37 weeks or 259 days) and term (greater than 37 weeks, up to and including 38 weeks and 3 days). Differences between myometrium from singleton and twin pregnancies were then examined by gestational age group (i.e. term singletons vs. term twins and preterm singletons vs. preterm twins).

Data were found to be non-parametric and significance was tested using the Mann Whitney U-test. In the third analysis, no grouping was done and all data was analyzed to compare the individual contractile measurements against neonatal birth weights, using Spearman's rank correlation test (correlation co-efficient is provided as *r*). For all analyses performed, a *P*-value of less than 0.05 was considered statistically significant.

## Results

### Baseline characteristics

Data were obtained on 48 twin pregnancies and 35 singletons. The mean data for maternal age, gestational age, parity and body mass index (BMI) in each group are shown in Table 1. There was no significant difference between any of the groups in terms of maternal age or BMI. Overall, there was a higher rate of primiparity in women with twin pregnancy compared to singleton pregnancy. However when sub-divided by gestational age group, there was no significant difference between groups. There was however a significant difference in term gestational age, with women with twins having a significantly shorter gestation than their singleton counterparts, despite limiting the singleton term samples to 269 days gestational age.

**Table 1:** Maternal characteristics of women with singleton and twin pregnancy.

	Combined			Term			Preterm		
Characteristic	Singleton (n = 35)	Twin (n = 48)	*P* value	Singleton (n = 17)	Twin (n = 18)	*P* value	Singleton (n = 18)	Twin (n = 30)	*P* value
**Gestational age (days)**	255 (2.5)	250 (2.3)	ns	266 (0.7)	262 (0.4)	<0.001*	245 (3.2)	243 (3.0)	ns
**Maternal age (years)**	32.0 (1.1)	31.6 (0.9)	ns	32.0 (1.6)	31.2 (1.6)	ns	32.2 (1.6)	31.9 (1.2)	ns
**BMI (Kg/m^2^)**	27.8 (1.1)	26.5 (0.8)	ns	25.1 (0.9)	26.7 (1.3)	ns	30.3 (1.9)	26.4 (1.1)	ns
**Parity (% primiparous)**	8 (22.9)	19.1 (51.4)	0.016*	3 (17.6)	8 (44.4)	ns	5 (27.8)	11 (36.6)	ns

Data is represented by means (± S.E.M.) or frequencies (counts, n and percentages).

* denotes significant difference (*P*<0.05) by unpaired t-test, or Chi Square (χ^2^) test (ns  =  not significant).

### Contractile properties

**Figure 1:**
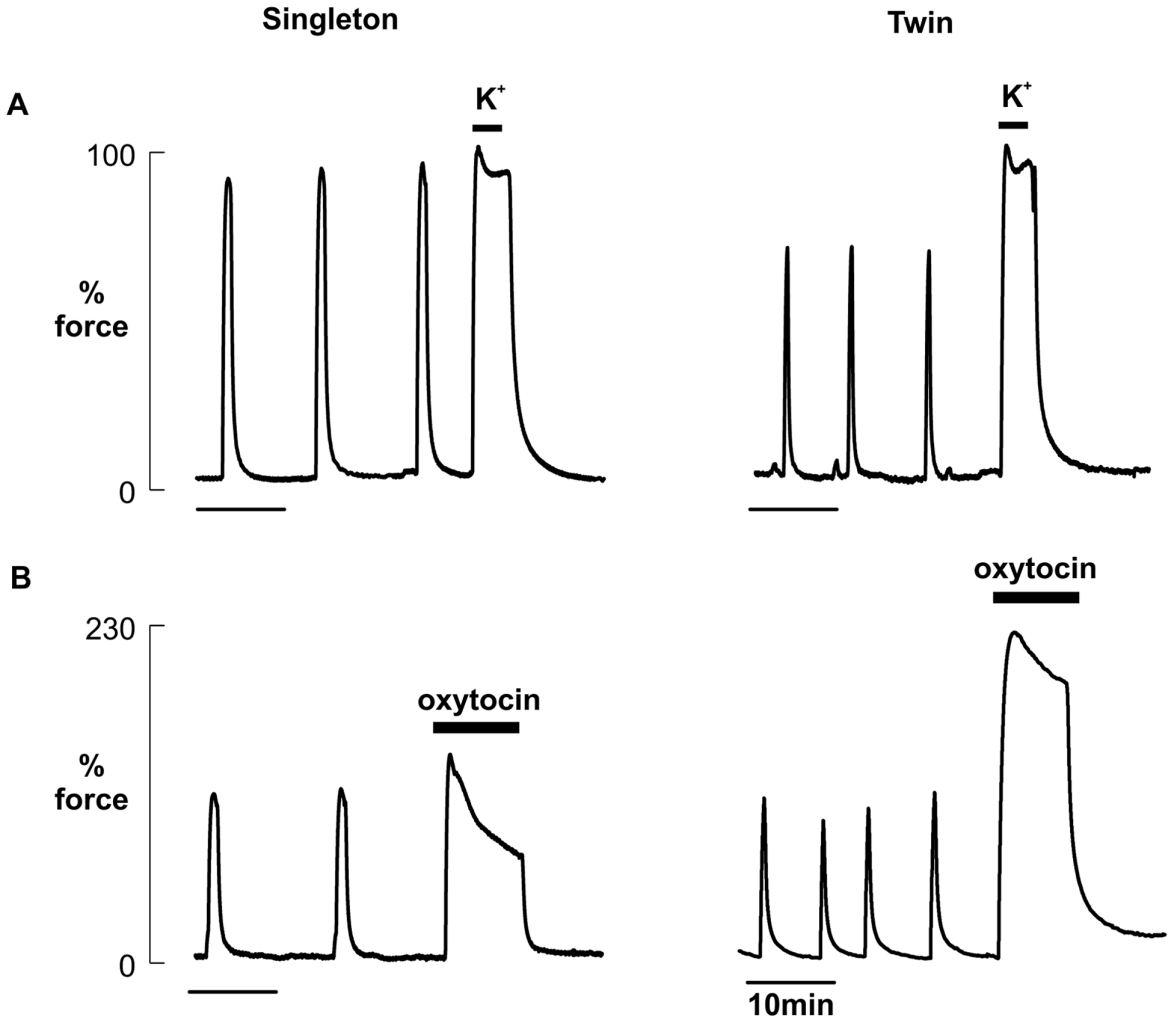
Contractile activity in human myometrium from singleton and twin pregnancy. Representative traces of spontaneous contractile activity of myometrium from singleton and twin pregnancies followed by (**A**) 2 min application of 40 mM KCl (K^+^) to depolarise the myometrium and (**B**) 20 min application of 10 nM oxytocin. In (A) contraction amplitude under high K was assigned 100%. The mean amplitude of control activity was then expressed as a percentage of high K and compared between groups. In (B), response to oxytocin was compared between singleton and twin samples by measuring the percentage increase in force amplitude of contraction under oxytocin where control activity equalled 100%.

Example experimental traces showing spontaneous contractile activity and responses to high K depolarization and oxytocin stimulation are shown in Figure 1A & B. Median data for amplitude, duration, frequency, and AUC (mean integral of force) for spontaneous activity are given in Table 2 for the singleton and twin pregnancy groups. Table 2 also shows the amplitude of spontaneous contractions relative to maximal (high K) force and AUC under high K and the percentage increase in force amplitude of contraction and AUC produced by oxytocin. When comparing the differences between all singleton and all twin pregnancies, there was a significant difference between the two groups in terms of duration and frequency of spontaneous contractions; the duration of contractions being significantly shorter (*P* = 0.004) and the frequency being significantly greater (*P* = 0.010) in the twin group. There was no difference between the two groups in terms of amplitude of contractions or AUC, either arising spontaneously, or with depolarised conditions or with oxytocin stimulation.

**Table 2:** Comparison of the contractile properties between myometrium from singleton and twin pregnancies.

Parameter	Singleton (n = 35)	Twin (n = 48)	*P* value
**Force amplitude (mN)**	3.37 (2.15–6.19)	2.21 (1.06–4.38)	ns
**Duration (min)**	1.02 (0.77–1.40)	0.79 (0.59–0.98)	*P* = 0.004*
**Frequency (no. 10 min^−1^)**	1.11 (0.71–1.6)	1.56 (0.99–2.16)	*P* = 0.010*
**Integral of force (AUC, a.u.)**	7.76 (4.68–13.17)	6.66 (3.23–16.19)	ns
**Force amplitude normalised to high K, %**	86.9 (65.2–99.4)	80.0 (48.4–89.8)	ns
**Integral of force under high K (AUC, a.u.)**	6.18 (2.31–10.59)	3.16 (2.13–6.18)	ns
**% increase in force amplitude under OT, (%)**	+41.4 (18.9–83.7)	+63.4 (35.1–118.0)	ns
**% increase in integral of force (AUC) under OT, (%)**	+522.6 (383.7–773.8)	+556.4 (176.9–1139.1)	ns

Data are represented by medians (IQR). * represents a significant difference (*P*<0.05) found by Mann Whitney U test (ns  =  not significant). High K is 40 mM to depolarize the myometrium. Contraction frequency measured over at least 4 contractions. OT; oxytocin, 10 nM.

Table 3 shows the data compared by both pregnancy type and gestational age. It was found that at term, twin pregnancies retained the duration and frequency differences found above; contractions were significantly shorter (*P* = 0.003), and more frequent (*P* = 0.014). In addition, it can be seen that the amplitude of spontaneous contractions was significantly more augmented by oxytocin (*P* = 0.027) in myometrium from twin compared to singleton pregnancies at term. However there was no significant difference in percentage increase in AUC under oxytocin stimulation. Comparison of the amplitude of spontaneous contraction normalised to depolarised conditions found the ratio to be significantly smaller in myometrium from twin pregnancies at term, compared to singletons at term (*P* = 0.034). There were no significant differences in amplitude or mean integral of force of spontaneous contractions, or under depolarised conditions, between myometrium from term twins and term singletons.

**Table 3:** Comparison of the contractile properties of myometrium from singleton and twin myometrium according to gestational age group.

	Term			Preterm		
Parameter	Singleton (n = 18)	Twin (n = 30)	*P* value	Singleton (n = 17)	Twin (n = 18)	*P* value
**Force amplitude (mN)**	2.89 (1.50–6.18)	1.95 (0.74–2.67)	ns	4.06 (1.89–6.66)	2.77 (1.22–4.82)	ns
**Duration (min)**	1.20 (0.90–1.41)	0.90 (0.66–0.10)	*P* = 0.003*	0.88 (0.63–1.42)	0.75 (0.57–0.98)	ns
**Frequency (no. 10 min^−1^)**	0.98 (0.74–1.24)	1.59 (0.85–2.30)	*P* = 0.014*	1.24 (0.71–1.93)	1.53 (1.02–2.16)	ns
**Integral of force (AUC, a.u.)**	6.16 (4.25–15.90)	6.66 (3.23–12.22)	ns	10.02 (5.76–12.82)	6.71 (3.27–18.54)	ns
**Force amplitude normalised to high K, (%)**	84.7 (65.2–94.3)	69.9 (45.0–85.9)	*P* = 0.034*	88.8 (64.4–102.0)	83.8 (71.2–93.5)	ns
**Integral of force under high K, (AUC, a.u.)**	5.72 (2.64–10.33)	2.52 (1.84–7.14)	ns	7.03 (2.26–11.74)	4.16 (2.43–5.74)	ns
**% increase in force amplitude under OT, (%)**	+30.9 (18.9–66.9)	+136.9 (48.1–274.8)	*P* = 0.027*	+59.9 (17.3–145.6)	+47.0 (32.5–73.6)	ns
**% increase in integral of force (AUC) under OT, (%)**	+522.61 ****(400.9–751.1)	+858.1 ****(244.1–1752.1)	ns	+508.1 (110.7–971.1)	+425.6 (250.9–922.2)	ns

Data are represented by medians (IQR) * represents a significant difference (*P*<0.05) found by Mann Whitney U-test (ns  =  not significant). High K is 40 mM to depolarize the myometrium. Contraction frequency measured over at least 4 contractions. OT; oxytocin, 10 nM.

When comparisons were made using myometrium from preterm singleton and twin pregnancies, there were no significant differences in any of the contractile properties measured, although frequency was still greater (but not significantly so) in the twin group (Table 3). In a sub-analysis of twin pregnancies, comparison of the response to oxytocin according to gestation group was made. We found oxytocin augmentation (increased force of contraction) in term twins was significantly greater than for preterm twins (amplitude was +136.9% term twins vs. +47% preterm twins, *P* = 0.022).

### Correlation between neonatal birth weight and contractile properties

**Figure 2:**
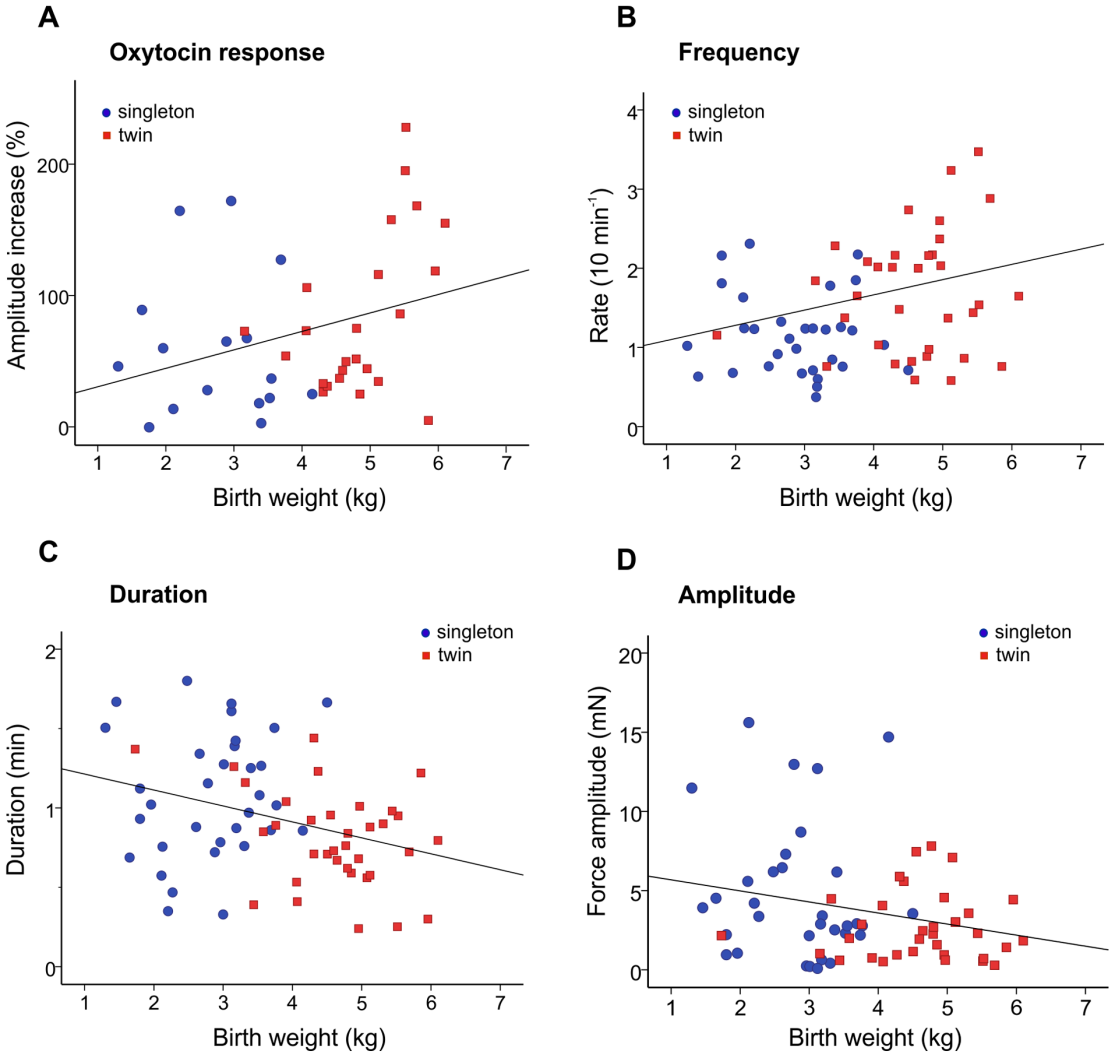
Contractile responses in relation to birth weight. Plots of the different contractile properties of strips of myometrium from women undergoing Caesarean section delivery with singleton (n = 35) or twin pregnancy (n = 48) against birth weight. (A) Response of the myometrium to 10 nM oxytocin, as percent increase relative to spontaneous contraction amplitude. (B) Frequency of spontaneous contractions (rate in 10 min). (C) Duration of spontaneous contractions (min). (D) Force amplitude of spontaneous contraction (mN). Increasing neonatal birth weight was significantly positively correlated with oxytocin response (A) and frequency of contraction (B), but negatively correlated with duration of contraction (C) and amplitude of contraction (D). In the case of twin pregnancy, birth weight is the combined weight of both babies. Data from myometrium from singleton pregnancies is denoted by blue circles and data from myometrium from twin pregnancies is denoted by red squares.

A plot of contractile properties from the myometrial samples was made against neonatal birth weight, which in the case of twins was the combined weight of both babies. There were no cases of polyhydramnios or oligohydramnios. As expected, the mean birth weight for the twin pregnancy group was significantly greater than singleton pregnancies; 4596 g (±132) twins, n = 48 versus 2814 g (±133) singletons, n = 35 (*P*<0.001). This difference also remained significant when comparing birth weights according to gestational age group (Term: 5178 g (±151) twins versus 3332g (±122) singletons; Preterm: 4247 g (±160) versus 2324g (±162) singletons, *P*<0.001 for both tests). Increasing neonatal birth weight was significantly positively correlated with oxytocin response (*r* = 0.287, *P* = 0.036, n = 40), Figure 2A. Thus, with increasing neonatal birth weight (and hence increased myometrial stretch) there was a greater increase in contraction augmentation by oxytocin. Frequency of contraction was also significantly positively correlated with neonatal birth weight, (*r* = 0.234, *P* = 0.027, n = 68, Figure 2B). Increasing neonatal birth weight however, was negatively correlated with the response to depolarised contractions (*r* = −0.263, *P* = 0.027, n = 54, not shown), and duration of contraction (*r* = −0.318, *P* = 0.004, n = 68, Figure 2C). There was also a trend towards decreased amplitude of spontaneous contractions with increasing neonatal birth weight (Figure 2D, r = −0.193, *P* = 0.115).

## Discussion

We demonstrate for the first time, the differences in myometrial contractility in singleton and twin pregnancies, across a range of gestational ages. We find that frequency of contractions and responses to oxytocin are increased in term twins compared to singletons. Furthermore, we show that contractile activity correlates with the increasing level of stretch, using neonatal birth weights as a marker of uterine stretch.

### Increased response to oxytocin

The amplitude of contraction with oxytocin was >2.5 times the amplitude of spontaneous contractions in term twin pregnancies, whereas in singleton pregnancies, the amplitude of contraction with oxytocin was only 1.3 times greater. Oxytocin stimulates contractions by a variety of mechanisms, including; opening of non-specific cation channels that cause membrane depolarisation, opening of L-type Ca channels and Ca entry, release of Ca from the sarcoplasmic reticulum [18] and decreasing Ca efflux mechanisms [19]. Stretch of myometrium stimulates oxytocin receptor (OTR) expression [20], [21], [22], [23]. Further, OTR expression is known to increase in labour in comparison to the non-labouring state [24] and oxytocin sensitivity is decreased in postdates pregnancies [25], [26]. It is not yet known whether OTR expression is greater in twin pregnancies compared to singleton pregnancies, although this could be a possible explanation for our findings and warrants further examination. Further support for this comes from our finding that increased stretch through increasing birth weight also increases the percentage change in contraction amplitude with oxytocin.

Whilst it could be speculated that increasing sensitivity to oxytocin is a mechanism of preterm labour in twin pregnancies, we have been unable to demonstrate enhanced contraction amplitude with oxytocin in preterm twin pregnancies, although it was significantly increased by term. This may be a consequence of the biopsies being from elective non-labouring women and hence the changes associated with labour, including OTR expression, may not have been established. Further studies with increased sample size and also of labouring myometrium would be needed to address this important point.

### Changes in contractile parameters

We have shown clear differences in the way myometrium in twin pregnancies contracts compared to singletons. This adds to previous *in vitro* studies showing contractility reflecting different clinical conditions, such as diabetes, obesity and postterm pregnancies [10], [26], [27]. Myometrium from twin pregnancies has contractions that are shorter but more frequent than myometrium from singleton pregnancies. These differences are again particularly apparent at term. The lack of difference between the groups in terms of mean integral of force can be explained by the singleton pregnancies having long lasting but less frequent contractions, whereas twins have frequent but short lasting contractions. The increase in frequency of contractions seen in term twins could be due to the shorter duration of contractions, meaning that less rest time is required between them, whereas myometrium from singleton pregnancies requires longer rest periods having had longer duration of contraction. It may also reflect different expression levels of ion channels in twin compared to singleton myometrium, as these govern firing of action potentials [28]. Clinically, contraction frequency is known to increase with ongoing labour [29], and increasing gestational age [30]. Pre-labour contraction frequency in preterm pregnancies is also increased [31].

### Role of uterine stretch

We show that stretch may play role in determining the frequency and duration of contraction, with increasing uterine distension from greater neonatal birth weight leading to shorter duration but increased frequency. There is little evidence of how duration of contractions may change in multiple pregnancy. Using a hands-on method to time duration, an increase with advancing labour was reported [32], however, more recent tocography assessment demonstrated that second stage contractions were actually two seconds shorter than contractions in the first stage [33].

A class of stretch activated potassium channels, TREK-1, have been identified in human myometrium [34] that were down regulated in labouring tissues [35]. Further, selective blockade of these channels leads to an increase in the frequency of contractions, suggesting a role in the maintenance of uterine quiescence. In murine models, it has been suggested that down-regulation of these channels induce the onset of delivery [36], although, the effect on the duration of contractions was not studied. While it is interesting to speculate about the role of TREK channels and the differences in frequency found, other channels, such as Ca-activated K channels [37] or cholride channels [38] which have been suggested to contribute to excitation and contractility in the myometrium, may also provide avenues for future investigation.

An increase in stretch also stimulates expression of oxytocin receptors and other contraction associated proteins, as mentioned above, and this could explain the increased responsiveness to oxytocin of term myometrium from women pregnant with twins compared to singletons. Increasing stretch of myometrial tissue leads to increasing expression of collagens, and increased focal adhesion between myocytes and extra-cellular matrix [9], [39], which may predispose to earlier activation of myometrium in twin pregnancies. It may also be that increasing uterine stretch leads to a decrease in the synchronicity of contractions in twins [40]. Such decreased synchronicity could lead to increased frequency, but lower amplitude spontaneous contractions, as we found, as only partial activation and regional conduction occurs. This would also explain why oxytocin, by synchronising contractile activity throughout the strip, has a much more pronounced effect on the term myometrium from women with twins compared to singletons.

However, there are data that mitigate against stretch being a unifying explanation for our findings: Data comparing myometrium from singleton and twin pregnancies showed that there was no difference in the expression of gap junction proteins, and that further mechanical stretch did not alter expression either [32], suggesting that propagation of action potentials is similar in singleton and twin pregnancies. Furthermore, recent work looking at uterine tension in multiple pregnancies found that whilst tension and intra-uterine pressures were greater in multiple pregnancies, it was not a causal factor in preterm labour [41], and there was no difference in uterine wall thickness between preterm and term pregnancies. Thus although increased stretch with multiple pregnancy is one candidate mechanism proposed to explain some of our novel observations on the contractile properties of myometrium from women with twin pregnancies, other mechanisms must be sought in future studies.

### Study Limitations

In this study we chose biopsies from elective sections from women not in labour, as this is more available than labouring tissue. Labouring tissues are also more varied, e.g. hours of labour, syntocinon administration, than non-labouring tissues and can therefore confound results when analysing contractility. While the elective sections have enabled us to obtain these novel data, any differences occurring once labour has started cannot be studied. This may have contributed to expected differences in preterm tissues of singletons and twins not being found in our study. Despite limiting the gestational age of term singletons accepted into the study, there was a significantly lower gestational age in the term twins compared to singletons. We consider however that this will have lead to an underestimate of the differences e.g. in frequency and oxytocin response, as both of these increased in twins with increasing gestation. Although obtaining data on 48 twins makes this the largest such study to date, it nevertheless means that the power of sub-analyses of the data is limiting. Future studies conducted over a longer period or involving more centres should be able to address this. Whilst we have considered stretch as a causative factor in the alteration in contractile activity, we recognise that other endocrine/paracrine factors may play a role in preterm labour and that of problems in labour such as obstruction may occur with twin deliveries [42].

### Conclusions

There are differences in the frequency and duration of contractions between myometrium from twin and singleton pregnancies, and the response to oxytocin is significantly greater in twin pregnancies. We have also shown that the degree of stretch from increasing neonatal birth weight affects these contractile properties. While some of these findings suggest a correlation with the increased frequency of preterm labour in twins, they also suggest future mechanistic studies on labouring and non-labouring tissues are needed.
